# Transition from metabolically healthy to unhealth status associated with risk of carotid artery plaque in Chinese adults

**DOI:** 10.1186/s12872-021-02279-w

**Published:** 2021-09-28

**Authors:** Tao Tan, Yiquan Zhou, Yanping Wan, Zhuping Fan, Renying Xu, Xiang Gao

**Affiliations:** 1 Department of Clinical Nutrition, Ren Ji Hospital South Campus, School of Medicine, Shanghai Jiao Tong University, Shanghai, 200127 China; 2 Shanghai Key Laboratory of Pediatrics Gastroenterology and Nutrition, Shanghai, China; 3 Department of Digestion, Ren Ji Hospital South Campus, School of Medicine, Shanghai Jiao Tong University, Shanghai, China; 4 Department of Nutrition Science, The Pennsylvania State University, State College, PA USA

**Keywords:** Metabolically healthy status, Metabolically unhealthy status, Carotid artery plaque (CAP), Adults

## Abstract

**Objective:**

We aimed to evaluate the association between the shift of metabolic status and future risk of carotid artery plaque (CAP) in community-based Chinese adults.

**Methods:**

The current study included 9836 Chinese adults (4085 males and 5751 females, mean age 35.8 years) with metabolically healthy status at baseline (2013). Metabolically healthy status was defined as no self-reported history of metabolic diseases and cancer, and normal blood pressure, fasting blood glucose, glycated hemoglobin A1c level, and lipid profiles. Metabolically unhealthy status was defined if any of the following metabolic abnormalities were confirmed twice during follow up: high blood pressure, impaired glucose regulation, high triglycerides, high total cholesterol, high low-density lipoprotein cholesterols, or low high-density lipoprotein cholesterols. The transition was confirmed if participants’ metabolic status shifted from baseline healthy to unhealthy status during follow up (2014–2018).

**Results:**

We have identified 133 incident cases of CAP during follow up. Compared to those who remained metabolically healthy, the transition to high blood pressure, high total cholesterol, and high low-density lipoprotein cholesterols, were associated with high risk of developing carotid artery plaque (Hazards ratios (HRs) ranged from 1.69 to 2.34; *p* < 0.05 for all). The transition to impaired glucose regulation, high total triglycerides, and low high-density lipoprotein cholesterols, were associated with high risk of carotid artery plaque only in participants with metabolically healthy overweight at baseline (HR ranged from 1.95 to 4.62; *p* < 0.05 for all).

**Conclusion:**

The transition from baseline metabolically healthy status to unhealth status was associated with high risk of incident CAP.

**Supplementary Information:**

The online version contains supplementary material available at 10.1186/s12872-021-02279-w.

## Introduction

The presence of atherosclerosis obviously contributes to many adverse events, such as ischemic stroke, myocardial infarction, and impairment of cognitive function [1–3]. Further, one-third cardiovascular related deaths are resulted from atherosclerosis, thus making it the leading cause of mortality worldwide [4].

Metabolic abnormalities such as high blood pressure, impaired glucose regulation, and dyslipidemia are considered as three major contributing factors to atherosclerosis. Overweight and obesity are considered as the source of metabolic abnormalities, however, a specific obesity phenotype, named as “metabolically healthy obesity” [5], aroused the arguments. Previous studies generated mixed results: some studies reported a similar risk of developing atherosclerosis for metabolically healthy obesity to those with metabolically healthy normal weight [6–10], while others found that metabolically healthy obesity was also associated with a high risk of developing atherosclerosis, relative to their normal-weight counterpart [11–19]. However, some concerns need to be addressed. First, the heterogeneity among study design: most of them were cross-sectional studies [6, 8–10, 12–19] and only 2 of them [7, 11] were cohort studies. Then, carotid intima-media thickness (IMT) [6, 8, 9, 11, 14, 16–18], coronary artery calcium score [7, 10, 12, 13, 15], and pulse wave velocity [19] were used to evaluate atherosclerosis. Finally, two studies [11, 14] were performed in men, one in women [18], one in patients with spinal cord injury [9], and others were in community population while sample size ranged from 38 [9] to 24,063 [10]. Existing evidences evaluating the association between baseline metabolically healthy obesity and CVD events also generated controversial results. For example, one national cohort study with long duration of follow up did not find the association between metabolically healthy obesity and ischemic stroke [20] while another national study reported metabolically healthy obesity increased the prevalence of stroke [21]. However, one meta-analysis only included prospective cohort studies reported that individuals with metabolically healthy obesity had an increased risk of stroke compared with metabolically healthy normal weight individuals (RR = 1.17, 95% CI: 1.11–1.23) [22]. Further, the effects of the transition from metabolically healthy status to metabolic abnormalities on the development of atherosclerosis were neglected among previous studies. Data regarding the effects of the transition on the development of atherosclerosis is limited. To the best of our knowledge, only one cohort study to date evaluated the association of transition from metabolically healthy to unhealthy status with the development of atherosclerosis [23]. The transition, but not baseline metabolically healthy status, was associated with high risk of atherosclerosis [23]. However, it has been doubtful to classify individuals with one metabolic risk factors, such as high blood pressure or impaired glucose regulation, as metabolically healthy status [5]. Further, different metabolic abnormalities might exert different effects on the developing atherosclerosis, thus it is appropriate to evaluate these risk factors (such as high blood pressure, impaired glucose regulation) separately rather than combining them together [24].

Therefore, we performed the current study in community-based Chinese population with metabolically healthy status and followed them for five years. CAP (carotid artery plaque), which was assessed by ultrasound B model annually, was served as an indicator for systematic atherosclerosis [25]. We hypothesized that the transition from baseline metabolically healthy status to metabolic abnormalities was associated with future risk of CAP.

## Subjects and methods

### Study population

All the participants were recruited from the Health Management Center in a teaching hospital from January 1, 2013 to December 31, 2018. The inclusion criteria were those: (1) who aged 18 years or more; (2) with normal blood pressure, fasting blood glucose (FBG), glycated hemoglobin A1c (HbA1c), lipid profile, and carotid artery B ultrasound assessment at baseline (2013). An initial recruitment resulted in an identification of 109,410 subjects. The total prevalence of metabolically healthy status was 31.2% and it was 44.4%, 17.7%, and 7.1% respectively for participants with normal weight (BMI < 24.0 kg/m^2^), overweight (24.0 kg/m^2^ ≤ BMI < 28.0 kg/m^2^), and obesity (BMI ≥ 28.0 kg/m^2^). Then, we performed a sequential recruitment. First, we excluded those with self-reported history of a series of metabolic diseases (n = 9622) and cancer (n = 29); Second, we excluded those with baseline metabolic abnormalities (n = 65,630); Third, we excluded those lost to follow up (2014–2018) (n = 13,378) and with missing data (n = 8083); Finally, we excluded those with low baseline BMI (≤ 18.4 kg/m^2^) (n = 2674); (6) aged 65 years or more because there were only 79 participants. (7) with CAP at baseline (n = 78); (8) with low eGFR < 60 ml/min/1.73m^2^ (n = 1). A total number of 9836 (4085 men and 5751 women, mean age of 35.8 ± 9.0 years) Chinese adults with metabolically healthy status (details were shown in Additional file 1: Fig. S1). Participants who were included in the study tended to be younger, had higher proportion of women and lower baseline BMI, level of HbA1c, FBG, and blood pressure, compared with those who were out of the study (Additional file 1: Table S1). The study protocol was approved by the Ethical Committee of Ren Ji Hospital, School of Medicine, Shanghai Jiao Tong University. As a de-identified secondary data analysis, patients’ consent was waived.

### Assessment of body weight, blood pressure and biochemical parameters

Body weight (to the nearest 0.5 kg) and height (to the nearest 0.5 cm) was measured in standing position without shoes and in light clothing, using an electronic scale (SK-CK, Shuang Jia Company, Shanghai, China). BMI was calculated by body weight (kg) divided by height square (m^2^). Blood pressure was measured twice using an automatic blood-pressure meter (HBP-9020, OMRON (China) Co., Ltd.) after participants were seated for at least 10 min. The average of two measurements was recorded for further analysis.

Venous blood samples were drawn and transfused into vacuum tubes containing EDTA in the morning after participants were fasted for at least six hours. An automatic analyzer (Roche 701 Bioanalyzer, Roche, UK) was used to measure FBG with the hexokinase/glucose-6-phosphate dehydrogenase method. The level of HbA1c was measured by high performance liquid chromatography, using the fully automated VARIANT™ II Hemoglobin Testing System (Bio-Rad, U.S). Total cholesterol (TC), triglycerides (TG), low-density lipoprotein cholesterols (LDL-C), and high-density lipoprotein cholesterols (HDL-C) were measured by an automatic biochemical analyzer (Roche 701 Bioanalyzer, Roche, UK). The estimated glomerular filatration rate (eGFR) was calculated using the Chronic Kidney Disease Epidemiology Collaboration 2-level race equation [26]. The concentration of high sensitivity CRP (hs-CRP) was measured by immune-tubidimetric method. All the measurements were completed in the Clinical Laboratory of our hospital.

### Assessment of history of metabolic diseases

The history of hypertension, diabetes/impaired glucose regulation, dyslipidemia, cardiovascular diseases (stroke, hemorrhage, coronary artery bypass grafting, stent surgery, and ischemic infarction), was collected via a self-report questionnaire.

### Assessment of CAP (outcome)

Ultrasound B-mode imaging was performed annually to detect CAP during five-year follow-up (Philips HDI 5000 ultrasound system equipped with a 7.5 MHz probe). Intima-media thickness was measured at the point approximately 1.5 cm away from the distal part of the bifurcation of common carotid artery. CAP is defined as a focal region with a thickness > 1.5 mm as measured from the media adventitia interface to the lumen-intima interface or as the presence of focal wall thickening that is at least 50% greater than that of the surrounding vessel wall [27].

### Definition of metabolically healthy status and unhealth status

Metabolically healthy status was defined as no self-reported history of metabolic diseases (hypertension, diabetes, dyslipidemia, and cardiovascular diseases) and cancer at baseline, and normal blood pressure, FBG, HbA1c, and lipid profiles. Metabolically unhealthy status was defined if any of the following metabolic abnormalities were confirmed twice during follow up: high blood pressure (systolic blood pressure ≥ 130 mmHg or diastolic blood pressure ≥ 80 mmHg) [28], impaired glucose regulation (FBG ≥ 5.6 mmol/L or HbA1c ≥ 5.7%) [29], high TC (≥ 5.72 mmol/L), high TG (≥ 1.7 mmol/L), high LDL-C (≥ 3.4 mmol/L), or low HDL-C (< 0.9 mmol/L in men and < 1.0 mmol/L in women). The transition was defined when participants’ metabolic status shifted from baseline healthy to unhealthy status during follow up (2014–2018). In the current study, the transition was considered as the exposure. In the secondary analysis, we evaluated the associated between baseline body weight status (normal weight vs. overweight) and the risk of developing metabolic abnormalities. Participants were classified into normal weight (18.5 ≤ BMI < 24.0 kg/m^2^) or overweight (BMI ≥ 24.0 kg/m^2^) groups based on the criteria for Chinese adults [30].

### Statistical analysis

Data were presented as mean ± standard deviation if it was in normal distribution and medium and quartile range if it was in abnormal distribution. We used non-paired student *t*-test if data were in normal distribution, and Chi-square analysis if data were in abnormal distribution, to analyze the differences between the two groups. We completed all statistical analyses by SAS version 9.4 (SAS Institute, Inc, Cary, NC). Formal hypothesis testing will be two-sided with a significant level of 0.05. Because the conversion was confirmed at least twice, we determined the person-time of follow-up for each participant from January 1, 2014 to either the first onset date of the conversion, or the end of follow-up (December 31, 2018), whichever came first.

First, we used the proportional Cox regression model to evaluate the association between baseline body weight (normal weight ***vs.*** overweight) and future risk of CAP. Then, we analyzed the association between the transition and future risk of CAP. We adjusted the potential confounders in different models: **model 1**, adjusting for age (y) and sex; **model 2**, adjusting for variables in model 1 and further adjusting for baseline eGFR (ml/min/1.73 m^2^) and hs-CRP (mg/L); and **model 3**, adjusting for variables in model 2 and further for baseline systolic blood pressure (mmHg), diastolic blood pressure (mmHg), FBG (mmol/L), HbA1c (%), TC (mmol/L), TG (mmol/L), LDL-C (mmol/L), HDL-C (mmol/L).

Likelihood ratio tests were conducted to examine statistical interactions between the transition and sex, and age, in relation to the CAP by comparing-2 log likelihood χ^2^ between nested models with and without the cross-product terms.

To test the robustness of the main results, we conducted two sensitivity analyses in model 2. First, we censored participants whose baseline level of hs-CRP was 10.0 mg/L or more [31]. Then, we censored participants who was confirmed with metabolic abnormalities once to lower the possibility of misclassification [32].

## Results

The current study included 9836 Chinese adults (4085 males and 5751 females) with metabolically healthy status at baseline, with a mean age of 35.8 ± 9.0 years old. Metabolically healthy status normal weight and overweight accounted for 76.4% (n = 7512) and 23.6% (n = 2324), respectively. Baseline characteristics were significantly different between the two groups except for total cholesterol (Table 1).

**Table 1:** The comparison of baseline characteristics according to baseline body weight in 9836 Chinese adults with metabolically healthy status

Variables	Normal weight (18.5 ≤ BMI ≤ 23.9 kg/m^2^)	Overweight (BMI ≥ 24.0 kg/m^2^)	P value
Sample number	7512	2324	–
Age, y	35.6 ± 8.9	36.3 ± 9.3	< 0.001
Sex, M/F, %	34.9/65.1	63.1/36.9	< 0.001
BMI, kg/m^2^	21.3 ± 1.5	25.8 ± 1.7	< 0.001
FBG, mmol/L	4.8 ± 0.4	4.8 ± 0.4	< 0.001
HbA1c, %	5.1 ± 0.3	5.2 ± 0.3	< 0.001
SBP, mmHg	108.2 ± 9.7	112.8 ± 8.7	< 0.001
DBP, mmHg	67.1 ± 6.9	69.6 ± 6.3	< 0.001
TC, mmol/L	4.4 ± 0.6	4.4 ± 0.6	0.08
TG, mmol/L	0.8 ± 0.3	1.0 ± 0.3	< 0.001
HDL-C, mmol/L	1.5 ± 0.3	1.3 ± 0.3	< 0.001
LDL-C, mmol/L	2.4 ± 0.5	2.6 ± 0.5	< 0.001
Hs-CRP, mg/L*	0.36 (0.2, 0.7)	0.7 (0.38, 1.34)	< 0.001
eGFR, ml/min/1.73m^2^	112.7 ± 12.3	109.8 ± 12.6	< 0.001

M, male; F, female; BMI, body mass index; FBG, fasting blood glucose; HbA1c, glycated hemoglobin A1c; SBP, systolic blood pressure; DBP, diastolic blood pressure; TC, total cholesterol; TG, total glycerides; HDL-C, high-density-lipoprotein cholesterol; LDL-C, low-density-lipoprotein cholesterol; hsCRP, high sensitivity C-reactive protein; eGFR, estimated glomerular filtration rate

Metabolically healthy status was defined no history of hypertension, diabetes mellitus, cardiovascular disease, dyslipidemia, and cancer, and normal blood pressure, FBG, HbA1c, and lipid profiles at baseline

*Abnormal distribution, data was represented by medium plus quartile range

**Fig. 1:**
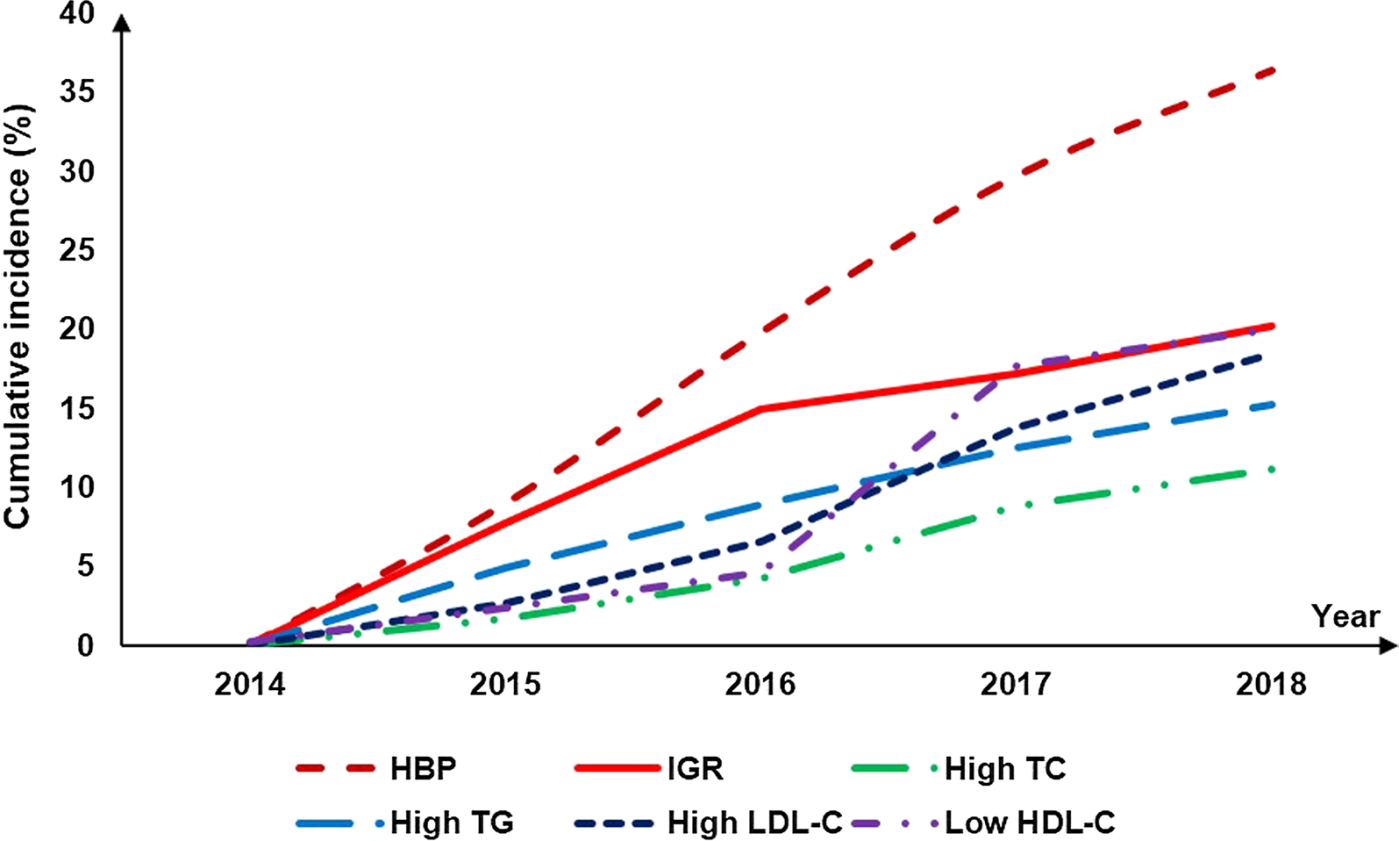
The cumulative proportion of different types of metabolic abnormalities during 5-year follow up. HBP, high blood pressure; IGR, impaired glucose regulation; TC, total cholesterol; TG, total triglycerides; LDL-C, low-density lipoprotein cholesterol; HDL-C, high-density lipoprotein cholesterol. Criteria: HBP: systolic blood pressure ≥ 130 mmHg and/or diastolic blood pressure ≥ 80 mmHg; IGR: fasting blood glucose ≥ 5.6 mmol/L and/or HbA1c ≥ 5.6%; high TC: ≥ 5.72 mmol/L; high TG: ≥ 1.7 mmol/L; high LDL-C: ≥ 3.4 mmol/L; low HDL-C: < 0.9 mmol/L in men and < 1.0 mmol/L in women

We did not find metabolically healthy overweight (BMI ≥ 24.0 kg/m^2^) was associated with future risk of CAP (HR = 0.92, 95%CI: 0.64, 1.32) (Additional file 1: Table S2). During 5-year follow up, the proportion who shifted from metabolically healthy to unhealthy status was 54.4% in participants with normal weight and 74.0% in participants with overweight at baseline. The cumulative proportion of developing HBP, IGR, high TC, high TG, high LDL-C, and low HDL-C was 36.4%, 20.1%, 11.2%, 15.3%, 18.4%, and 20.0%, respectively (Fig. 1). Baseline metabolically healthy overweight was associated with high risk of developing high blood pressure [hazards ratio (HR) = 1.23; 95% confidence interval (CI): 1.14, 1.33] and impaired glucose regulation (HR = 1.15; 95% CI: 1.04, 1.28) in fully adjusted model, compared to metabolically healthy normal weight group (Table 2). Baseline healthy overweight was associated with high TC, high TG, high LDL-C, and low HDL-C in age- and sex- adjusted model, however, the association lost significance after further adjusting baseline blood pressure, FBG, HbA1c, and lipid profile except a marginal association between metabolically healthy overweight and high TG (HR = 1.12, 95% CI: 1.001, 1.25, *p* = 0.046) (Table 2).

**Table 2:** Risk of developing different metabolic abnormalities during follow-up, according to baseline body weight status in 9836 Chinese adults who were metabolically healthy

Outcome	Metabolically healthy normal weight	Metabolically healthy overweight
	(BMI < 24.0 kg/m^2^)	(BMI ≥ 24.0 kg/m^2^)
*HBP*		
Number of participants	7512	2324
Incident case	2400	1177
Model 1	**Ref (1.0)**	1.50 (1.4, 1.61)
Model 2	**Ref (1.0)**	1.49 (1.39, 1.6)
Model 3	**Ref (1.0)**	1.23 (1.14, 1.33)
*IGR*		
Number of participants	7512	2324
Incident case	1394	594
Model 1	**Ref (1.0)**	1.36 (1.24, 1.51)
Model 2	**Ref (1.0)**	1.31 (1.2, 1.49)
Model 3	**Ref (1.0)**	1.15 (1.04, 1.28)
*High TC*		
Number of Participants	7512	2,324
Incident case	836	266
Model 1	**Ref (1.0)**	1.08 (0.94, 1.25)
Model 2	**Ref (1.0)**	1.07 (0.93, 1.24)
Model 3	**Ref (1.0)**	1.03 (0.89, 1.19)
*High TG*		
Number of participants	7512	2324
Incident case	908	595
Model 1	**Ref (1.0)**	1.79 (1.6, 1.99)
Model 2	**Ref (1.0)**	1.77 (1.59, 1.97)
Model 3	**Ref (1.0)**	1.12 (1.001, 1.25)
*High LDL-C*		
Number of participants	7512	2324
Incident case	1264	550
Model 1	**Ref (1.0)**	1.31 (1.18, 1.45)
Model 2	**Ref (1.0)**	1.3 (1.17, 1.44)
Model 3	**Ref (1.0)**	0.99 (0.89, 1.11)
*Low HDL-C*		
Number of participants	7512	2324
Incident case	371	238
Model 1	**Ref (1.0)**	1.78 (1.5, 2.1)
Model 2	**Ref (1.0)**	1.75 (1.48, 2.08)
Model 3	**Ref (1.0)**	0.99 (0.83, 1.18)

If the number "1" is not included in the confidence interval, it means significant

Model 1: adjusting for age and sex. Model 2: adjusting for variables in model 1 and estimated glomerular filtration rate (ml/min/1.73 m^2^), and high sensitivity C-reactive protein (mg/L). Model 3: adjusting for variables in model 2 and systolic blood pressure (mmHg), diastolic blood pressure (mmHg), FBG (mmol/L), HbA1c (%), TC (mmol/L), TG (mmol/L), LDL-C (mmol/L), HDL-C (mmol/L)

HBP, high blood pressure; FBG, fasting blood glucose; HbA1c, glycated hemoglobin A1c; IGR, impaired glucose regulation; TC, total cholesterol; TG, total triglycerides; LDL-C, low-density lipoprotein cholesterol; HDL-C, high-density lipoprotein cholesterol

Definition: Metabolically healthy was defined as participants without history of high blood pressure, diabetes mellitus, cardiovascular disease, dyslipidemia, and cancer but with normal blood pressure, FBG, HbA1c, TC, TG, LDL-C, and HDL-C. Criteria for metabolic abnormality: HBP (systolic blood pressure ≥ 130 mmHg or diastolic blood pressure ≥ 80 mmHg); IGR (FBG ≥ 5.6 mmol/L or HbA1c ≥ 5.7%); high TC, ≥ 5.72 mmol/L; high TG, ≥ 1.7 mmol/L; high LDL-C, ≥ 3.4 mmol/L; low HDL-C, < 0.9 mmol/L in men and < 1.0 mmol/L in women

We have identified 133 incident cases of CAP during follow up. Compared to those who remained metabolically healthy status, we found that transition to high blood pressure, high TC, and high LDL-C during follow up were associated a high likelihood of developing CAP, regardless of baseline body weight status (Table 3). When considering the transition to impaired glucose regulation, high TG, and low HDL-C, the association only remained in participants with baseline metabolically healthy overweight (Table 3).

**Table 3:** Risk of incident carotid artery plaque by baseline body weight status and transition to metabolic abnormalities in 9836 Chinese adults

Models	Metabolically healthy normal weight		Metabolically healthy overweight	
	(n = 7512, BMI < 24.0 kg/m^2^)		(n = 2324, BMI ≥ 24.0 kg/m^2^)	
	Stable	Transition to HBP	Stable	Transition to HBP
Number of participants	5112	2400	1147	1177
CAP case	35	50	14	34
Model 1	**Ref (1.0)**	1.64 (1.05, 2.55)	1.24 (0.66, 2.31)	2.35 (1.45, 3.83)
Model 2	**Ref (1.0)**	1.62 (1.04, 2.53)	1.22 (0.62, 2.29)	2.36 (1.45, 3.85)
Model 3	**Ref (1.0)**	1.69 (1.06, 2.68)	1.18 (0.62, 2.23)	2.29 (1.35, 3.87)

	Stable	Transition to IGR	Stable	Transition to IGR
Number of participants	6118	1394	1730	594
CAP case	56	29	24	24
Model 1	**Ref (1.0)**	1.22 (0.78, 1.93)	1.18 (0.73, 1.91)	2.17 (1.34, 3.53)
Model 2	**Ref (1.0)**	1.2 (0.76, 1.91)	1.19 (0.73, 1.93)	2.15 (1.33, 3.51)
Model 3	**Ref (1.0)**	1.17 (0.73, 1.87)	1.11 (0.67, 1.81)	1.95 (1.15, 3.3)

	Stable	Transition to high TC	Stable	Transition to high TC
Number of participants	6676	836	2058	266
CAP case	61	24	36	12
Model 1	**Ref (1.0)**	2.26 (1.39, 3.65)	1.45 (0.95, 2.19)	2.7 (1.45, 5.03)
Model 2	**Ref (1.0)**	2.25 (1.38, 3.66)	1.45 (0.96, 2.2)	2.71 (1.46, 5.05)
Model 3	**Ref (1.0)**	2.04 (1.22, 3.43)	1.33 (0.87, 2.05)	2.34 (1.21, 4.53)

	Stable	Transition to high TG	Stable	Transition to high TG
Number of participants	6604	908	1729	595
CAP case	69	16	27	21
Model 1	**Ref (1.0)**	1.23 (0.71, 2.12)	1.14 (0.73, 1.79)	2.37 (1.44, 3.9)
Model 2	**Ref (1.0)**	1.23 (0.72, 2.13)	1.15 (0.74, 1.81)	2.37 (1.44, 3.92)
Model 3	**Ref (1.0)**	1.23 (0.69, 2.2)	1.08 (0.68, 1.71)	2.27 (1.30, 3.95)

	Stable	Transition to high LDL-C	Stable	Transition to high LDL-C
Number of participants	6248	1264	1774	550
CAP case	51	34	31	17
Model 1	**Ref (1.0)**	2.26 (1.46, 3.5)	1.59 (1.01, 2.49)	2.39 (1.38, 4.16)
Model 2	**Ref (1.0)**	2.27 (1.47, 3.51)	1.6 (1.02, 2.51)	2.4 (1.38, 4.17)
Model 3	**Ref (1.0)**	1.95 (1.20, 3.17)	1.52 (0.95, 2.42)	2.03 (1.11, 3.73)

	Stable	Transition to low HDL-C	Stable	Transition to low HDL-C
Number of participants	7403	109	2252	72
CAP case	83	2	45	3
Model 1	**Ref (1.0)**	1.68 (0.41, 6.83)	1.37 (0.95, 1.98)	4.55 (1.43, 14.53)
Model 2	**Ref (1.0)**	1.65 (0.41, 6.71)	1.37 (0.95, 1.99)	4.51 (1.41, 14.4)
Model 3	**Ref (1.0)**	1.61 (0.39, 6.69)	1.27 (0.86, 1.87)	4.62 (1.4, 15.22)

If the number "1" is not included in the confidence interval, it means significant

Model 1: adjusting for age and sex. Model 2: adjusting for variables in model 1 and estimated glomerular filtration rate (ml/min/1.73 m^2^), and high sensitivity C-reactive protein (mg/L). Model 3: adjusting for variables in model 2 and systolic blood pressure (mmHg), diastolic blood pressure (mmHg), FBG (mmol/L), HbA1c (%), TC (mmol/L), TG (mmol/L), LDL-C (mmol/L), HDL-C (mmol/L)

HBP, high blood pressure; FBG, fasting blood glucose; HbA1c, glycated hemoglobin A1c; IGR, impaired glucose regulation; TC, total cholesterol; TG, total triglycerides; LDL-C, low-density lipoprotein cholesterol; HDL-C, high-density lipoprotein cholesterol

Definition: Metabolically healthy was defined as participants without history of high blood pressure, diabetes mellitus, cardiovascular disease, dyslipidemia, and cancer but with normal blood pressure, FBG, HbA1c, TC, TG, LDL-C, and HDL-C. Criteria for metabolic abnormality: HBP (systolic blood pressure ≥ 130 mmHg or diastolic blood pressure ≥ 80 mmHg); IGR (FBG ≥ 5.6 mmol/L or HbA1c ≥ 5.7%); high TC, ≥ 5.72 mmol/L; high TG, ≥ 1.7 mmol/L; high LDL-C, ≥ 3.4 mmol/L; low HDL-C, < 0.9 mmol/L in men and < 1.0 mmol/L in women

We found that the association between the transition to metabolic abnormalities and future risk of CAP was modified by age (all *p* < 0.01), but not sex (Additional file 1: Table S3). The association between the transition to metabolic abnormalities and future risk of CAP was more pronounced in participants aged 34 years or more (Additional file 1: Table S3). Excluding participants whose baseline level of hs-CRP was 10 mg/L did not substantially change the association (Additional file 1: Table S4). Excluding participants who were confirmed with metabolic abnormalities once during follow up, the association remained a similar trend. However, the association lost significance in some groups because of small incident cases of CAP (Additional file 1: Table S5).

The risk of CAP increased simultaneously with the increase of metabolic abnormalities, compared with those remained metabolically healthy status (*p* trend < 0.001, Additional file 1: Table S6).

## Discussion

In the current study including 9836 Chinese adults with metabolically healthy status, we found that the transition to metabolic abnormalities were associated with high risk of developing CAP, after adjusting a series of traditional risk factors, including blood pressure, FBG, HbA1c, lipid profiles, eGFR, and hs-CRP. If the participants were overweight at baseline but remained metabolically healthy status, the risk of CAP was similar with those whose baseline body weight were normal and remained metabolically healthy during the follow up.

Our results did not support the hypothesis that baseline metabolically healthy obesity was associated with high risk of CAP. Limited to studies in general population with large sample size (n ≥ 1000), three cross-sectional studies have been performed in Korean adults in which atherosclerosis was assessed by coronary artery calcium score [10, 12, 13]. One reported metabolically healthy status was more closely associated with atherosclerosis than obesity [10] while another two found that participants with metabolically healthy obesity had a higher prevalence of atherosclerosis than their metabolically healthy normal weight counterparts [12, 13]. Our results were consistent with one cross-sectional study in which atherosclerosis was assessed by the same method (ultrasound B model) [6]. They found that participants with normal weight and metabolic abnormalities, but not those with overweight but with metabolically healthy, was associated with high prevalence of atherosclerosis [6]. As for cohort study, Kim et al. [11] reported baseline metabolically healthy overweight (HR = 1.24; 95% CI: 1.12, 1.38) and obesity (HR = 1.54; 95% CI: 1.38, 1.72) were associated with CAP in 6543 men with a median follow up of 4.2 years. However, as the author acknowledged that including only men were in the study was obvious a limitation. The different definition of metabolically healthy obesity, in combination with ethnicity, population, and statistical method, was blamed for the discrepancies among the studies [15].

One possible explanation was that metabolically healthy obesity was defined based on BMI and BMI was often blamed for its shortcomings of not distinguishing body fat from lean body mass. Metabolically healthy obese might have a better adipose tissue function and less ectopic fat storage than persons with both obesity and metabolic abnormalities. A mendelian randomization study using summarized data including 24 metabolic phenotypes from 10 consortiums reported that the odds ratios and 95% CIs of a 1-SD increase in body fat mass ranged from 1.11 to 1.41 (all *p* < 0.05) for type 2 diabetes mellitus, hypertension, coronary artery disease, myocardial infarction, and ischemic stroke [33]. Another mendelian study performed in 367,703 UK Biobank participants reported that fat mass index associated stronger with aortic valve stenosis than BMI (1.06 for per 1 kg/m^2^ vs. 1.46 for fat mass index) [34]. Another reason for heterogeneity was lack of consensus of definition of metabolically healthy obesity [5].

Another critical point was the transition from baseline metabolically healthy to metabolic abnormalities, which was previously neglected among previous studies. Chang et al. [13] also pointed out the association between baseline metabolically healthy obesity and atherosclerosis could be mediated by components of metabolic abnormalities. Further, the Nurse’s Health Study reported that 84% of women with obesity and 68% of women with normal-weight were confirmed with metabolic abnormalities after 20 years [24], which indicated that metabolically healthy was unstable and eventually transited to unhealthy status. Lin et al. have performed a very interesting cohort study in 6220 Chinese adults with metabolically healthy (≤ 2 components of metabolic syndrome based on ATP III). Transition to metabolic abnormalities was associated with an odd ratio of 2.52 (95%CI: 1.89, 3.36) for the risk of atherosclerosis compared with their normal weight counterparts [23]. Similar with Lin’s study, we also confirmed that the transition was associated with future risk of atherosclerosis. In addition, we found that different types of metabolic abnormalities differed in their effects on the development of atherosclerosis. Abnormalities of blood pressure, TC, and LDL-C were associated a high likelihood of developing atherosclerosis in both metabolically healthy overweight and normal weight groups while the occurrence of abnormalities of glucose regulation, TG, and HDL-C were associated with atherosclerosis only in those who were overweight at baseline.

It is not surprising that we found that age modified the association between the transition and atherosclerosis. In other words, the association remained in elder (≥ 34 years), but not younger participants. Previous studies have proved that the prevalence of both total atherosclerotic related cardiovascular diseases [35] and peripheral artery diseases increased with age [36]. We did not find an interaction of sex with the association, which was consistent with previous studies [13, 15].

The strengthens of the current study includes more stricter definition of metabolically healthy status, a prospective study design, and a fully adjustment of traditional risk factors including blood pressure, FBG, HbA1c, and lipid profile though they were in normal range. However, some limitations must be addressed. First, the history of metabolic diseases was self-reported, which was lower than national prevalence. We thus excluded those with abnormalities at baseline, which could reduce the possibility of misclassification. Second, information about medical intervention, diet, and physical activities was deficient. Medical treatment such as aspirin was deficient, which was known to be associated with the development of atherosclerosis [37]. The information on lipid-lowering drugs such as statins was also deficient, which could lead to misclassification of dyslipidemia and was associated with cardiovascular diseases [38]. We cannot exclude the possibility that some participants were on diet and this might distract the results. Similarly, we did not collect information on waist circumference. Waist circumference is better to predict visceral adipose tissue than BMI and it is closer associated with CAP [39], however, BMI is the most widely used parameter to diagnose overweight and obesity. Third, all the participants were recruited from those who underwent health checkup in our hospital. It accounted for a small proportion of the total residents; thus, the generalizability of study population was limited. Fourth, the ideal time for fasting when drawing blood might be 8–10 h. However, it was shorter (at least 6 h fasting) in the current study and this might have some effects on the value of fasting blood glucose and lipid profiles. Finally, the number of incident CAP cases was small in the current study. Multiple-center based cohort studies with a face-to-face interview about history of diseases and medical information were needed to duplicate our results.

## Conclusions

The transition from baseline metabolically healthy to metabolic abnormalities was associated with high risk of incident atherosclerosis. Six out of ten participants developed at least one type of metabolically abnormalities during follow up, and the proportion was higher in participants with overweight, compared with those with normal weight at baseline. Early interventions focusing on both baseline body weight and the occurrence of metabolic abnormalities are meaningful to reduce atherosclerosis related disability and mortality.

## Supplementary Information

**Additional file 1.** The supplemental figure and tables.

## Data Availability

The SAS code and data that support the findings of this study on GitHub website: https://github.com/xurenying7465/xurenying7465.
