# Supplementary material for: Transition from metabolically healthy to unhealth status associated with risk of carotid artery plaque in Chinese adults
Source: BMC Cardiovasc Disord. 2021 Sep 28;21:469. doi: 10.1186/s12872-021-02279-w (PMC8477551; doi:10.1186/s12872-021-02279-w)
Supplement: Supplementary file 1 — Additional file 1. The supplemental figure and tables. [file 12872_2021_2279_MOESM1_ESM.docx]

**
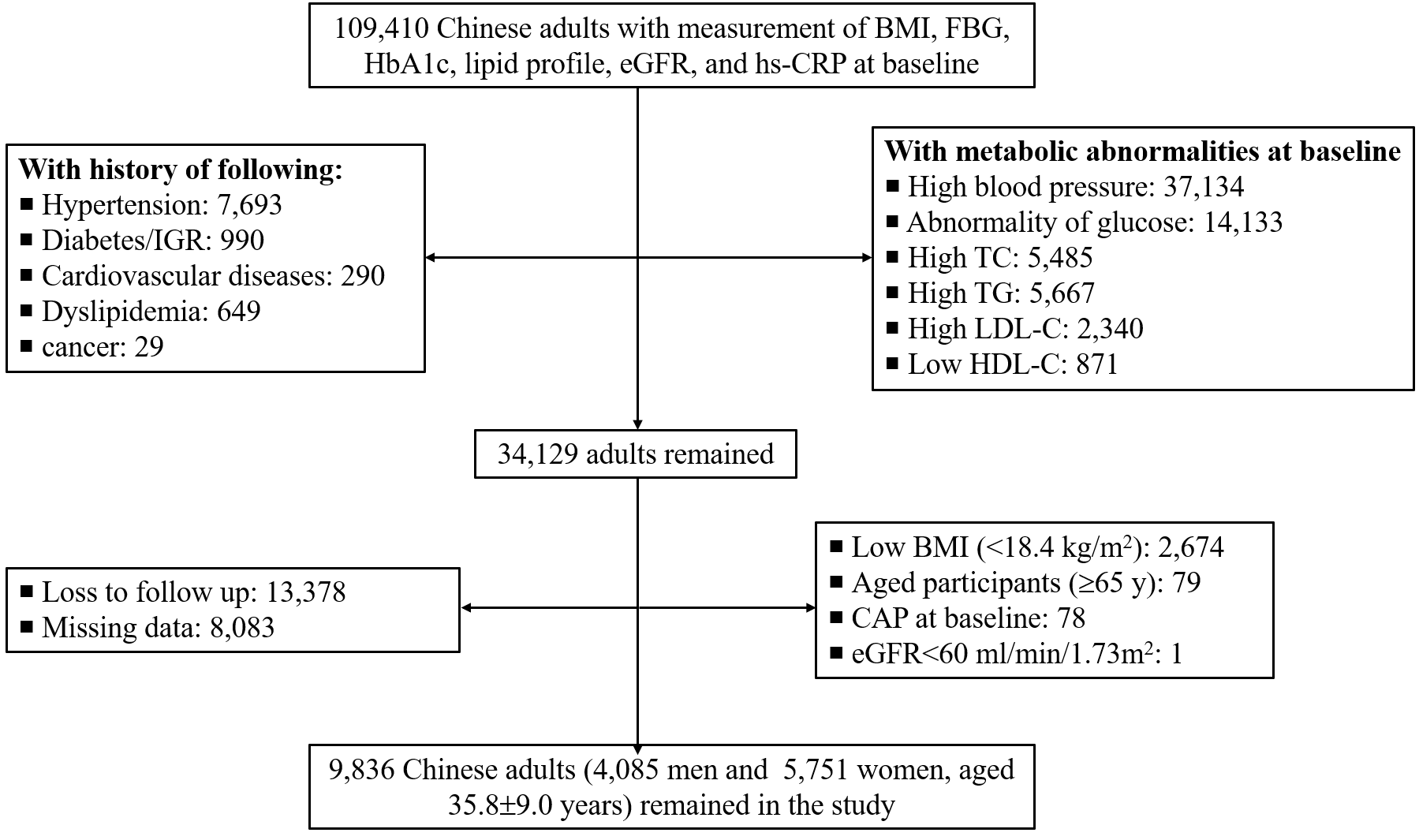
**

**Supplemental Figure 1.** The process of sample recruitment.

1. **Abbreviation**: **BMI**, body mass index; **FBG**, fasting blood glucose; **HbA1c**, glycated hemoglobin A1c; eGFR, estimated glomerular filtration rate; **hs-CRP**, high sensitivity C-reactive protein; **IGR**, impaired glucose regulation; **TC**, total cholesterol; **TG**, total triglycerides; **LDL-C**, low density lipoprotein cholesterol; **HDL-C**, high density lipoprotein cholesterol; **CAP,** carotid artery plaque.

2. **Criteria**: high blood pressure (systolic blood pressure≥130 mmHg or diastolic blood pressure≥80 mmHg); abnormality of glucose (FBG ≥5.6 mmol/L or HbA1c≥5.7%); high TC (TC≥5.72mmol/L); high TG (TG≥1.7 mmol/L); high LDL-C (LDL-C≥3.4mmol/L); low HDL-C (HDL-C<0.9 mmol/L in men and <1.0 mmol/L in women).

**Supplemental Table 1**. Baseline characteristics between participants in and out of the study

| Variables | In the study | Out of the study | P value |
| --- | --- | --- | --- |
| Sample number | 9,836 | 99,574 | -- |
| Age, y | 35.8±9.0 | 43.6±13.0 | <0.001 |
| Sex, M/F, % | 41.5/58.5 | 57.0/43.0 | <0.001 |
| BMI, kg/m^2^ | 22.3±2.5 | 24.0±3.4 | <0.001 |
| FBG, mmol/L | 4.8±0.4 | 5.3±1.1 | <0.001 |
| HbA1c, % | 5.1±0.3 | 5.5±0.7 | <0.001 |
| SBP, mmHg | 109.3±9.6 | 123.3±17.6 | <0.001 |
| DBP, mmHg | 67.7±6.9 | 76.2±11.5 | <0.001 |
| TC, mmol/L | 4.4±0.6 | 5.0±0.9 | <0.001 |
| TG, mmol/L | 0.9±0.3 | 1.6±1.3 | <0.001 |
| HDL-C, mmol/L | 1.5±0.3 | 1.4±0.4 | <0.001 |
| LDL-C, mmol/L | 2.4±0.5 | 2.9±0.8 | <0.001 |
| Hs-CRP, mg/L* | 0.4 (0.21, 0.83) | 0.65 (0.35, 1.3) | <0.001 |
| eGFR, ml/min/1.73m^2^ | 112.1±12.4 | 104.2±14.8 | <0.001 |

**Note:**

**1.** **Abbreviation**: **M**, male; **F**, female; **BMI**, body mass index; **FBG**, fasting blood glucose; **HbA1c**, glycated hemoglobin A1c; **SBP**, systolic blood pressure; **DBP**, diastolic blood pressure; **TC**, total cholesterol; **TG**, total glycerides; **HDL-C**, high-density-lipoprotein cholesterol; **LDL-C**, low-density-lipoprotein cholesterol; **hs-CRP**, high sensitivity C-reactive protein; **eGFR**, estimated glomerular filtration rate.

**2.** *, abnormal distribution, data was represented by medium plus quartile range.

**Supplemental Table 2**. Risk of incident carotid artery plaque by baseline body weight status in 9,836 participants with metabolically healthy status

| Model | Metabolically healthy normal weight  (BMI<24.0 kg/m^2^) | Metabolically healthy normal weight  (BMI<24.0 kg/m^2^) |
| --- | --- | --- |
| Participants, No (%) | 7,427 (98.9) | 2,276 (97.9) |
| CAP Case, No (%) | 95 (1.1) | 48 (2.1) |
| Model 1 | **Ref (1.0)** | 0.95 (0.62, 1.43) |
| Model 2 | **Ref (1.0)** | 0.93 (0.65, 1.32) |
| Model 3 | **Ref (1.0)** | 0.92 (0.64, 1.31) |

**Note:**

**1.** **Model 1**: adjusting for age and sex. **Model 2**: adjusting for variables in model 1 and estimated glomerular filtration rate (ml/min/1.73m^2^), and high sensitivity C-reactive protein (mg/L). **Model 3**: adjusting for variables in model 2 and systolic blood pressure (mmHg), diastolic blood pressure (mmHg), FBG (mmol/L), HbA1c (%), TC (mmol/L), TG (mmol/L), LDL-C (mmol/L), HDL-C (mmol/L).

**2. Abbreviation**: **HBP**, high blood pressure; **FBG**, fasting blood glucose; **HbA1c**, glycated hemoglobin A1c; **IGR**, impaired glucose regulation; **TC**, total cholesterol; **TG**, total triglycerides; **LDL-C**, low-density lipoprotein cholesterol; **HDL-C**, high-density lipoprotein cholesterol.

**3.** **Definition**: Metabolically healthy was defined as participants without history of high blood pressure, diabetes mellitus, cardiovascular disease, dyslipidemia, and cancer but with normal blood pressure, FBG, HbA1c, TC, TG, LDL-C, and HDL-C. **Criteria for metabolic abnormality**: **HBP** (systolic blood pressure≥130 mmHg or diastolic blood pressure≥80 mmHg); **IGR** (FBG ≥5.6 mmol/L or HbA1c≥5.7%); high TC, ≥5.72mmol/L; high TG, ≥1.7 mmol/L; high LDL-C, ≥3.4mmol/L; low HDL-C, <0.9 mmol/L in men and <1.0 mmol/L in women.

**Supplemental Table 3**. Risk of incident carotid artery plaque by baseline body weight status and transition to metabolic abnormalities: sub-grouped by sex and medium of age

| Group | Metabolically healthy normal weight  (BMI<24.0 kg/m^2^) | | Metabolically healthy overweight  (BMI≥24.0 kg/m^2^) | | P interaction |
| --- | --- | --- | --- | --- | --- |
|  | Stable | Transition to HBP | Stable | Transition to HBP | 0.11 |
| Men | **Ref (1.0)** | 1.29 (0.71, 2.32) | 1.21 (0.59, 2.51) | 1.02 (0.85, 3.1) |  |
| Women | **Ref (1.0)** | 2.35 (1.12, 4.93) | 0.69 (0.16, 3.09) | 4.11 (1.75, 9.63) |  |
| <34 years | **Ref (1.0)** | 6.06 (0.56, 64.8) | 4.25 (0.24, 75.7) | 3.80 (0.19, 78.1) | <0.001 |
| ≥34 years | **Ref (1.0)** | 2.13 (1.33, 3.42) | 1.18 (0.61, 2.29) | 2.54 (1.48, 4.34) |  |
|  | Stable | Transition to IGR | Stable | Transition to IGR |  |
| Men | **Ref (1.0)** | 1.03 (0.54, 1.95) | 1.04 (0.58, 1.86) | 1.80 (0.94, 3.43) | 0.57 |
| Women | **Ref (1.0)** | 1.35 (0.65, 2.81) | 1.19 (0.47, 3.01) | 2.52 (1.03, 6.18) |  |
| <34 years | **Ref (1.0)** | 20.0 (1.91, 209) | 5.87 (0.47, 73.8) | N/A | 0.001 |
| ≥34 years | **Ref (1.0)** | 1.23 (0.76, 2.01) | 0.99 (0.59, 1.65) | 2.05 (1.21, 3.48) |  |
|  | Stable | Transition to high TC | Stable | Transition to high TC |  |
| Men | **Ref (1.0)** | 3.03 (1.54, 5.95) | 1.28 (0.76, 2.18) | 2.86 (1.31, 6.25) | 0.35 |
| Women | **Ref (1.0)** | 1.33 (0.6, 2.92) | 1.56 (0.75, 3.24) | 1.61 (0.45, 5.69) |  |
| <34 years | **Ref (1.0)** | 104 (8.16, 1328) | N/A | 152 (8.79, 2660) | 0.1 |
| ≥34 years | **Ref (1.0)** | 1.9 (1.11, 3.27) | 1.27 (0.82, 1.96) | 2.16 (1.06, 4.4) |  |
|  | Stable | Transition to high TG | Stable | Transition to high TG |  |
| Men | **Ref (1.0)** | 0.99 (0.47, 2.12) | 1.06 (0.61, 1.86) | 1.75 (0.89, 3.44) | 0.29 |
| Women | **Ref (1.0)** | 1.79 (0.71, 4.52) | 1.004 (0.43, 2.33) | 5.0 (1.93, 13.0) |  |
| <34 years | **Ref (1.0)** | 17.7 (2.05, 152) | N/A | 31.1 (2.32, 416) | 0.005 |
| ≥34 years | **Ref (1.0)** | 1.04 (0.56, 1.93) | 1.02 (0.64, 1.64) | 1.96 (1.09, 3.5) |  |
|  | Stable | Transition to high LDL-C | Stable | Transition to high LDL-C |  |
| Men | **Ref (1.0)** | 2.18 (1.16, 4.13) | 1.33 (0.74, 2.40) | 2.40 (1.19, 5.03) | 0.47 |
| Women | **Ref (1.0)** | 1.82 (0.93, 3.57) | 1.44 (0.90, 2.32) | 2.26 (1.01, 5.03) |  |
| <34 years | **Ref (1.0)** | 60.1 (4.69, 769) | N/A | 85.1 (4.46, 1623) | 0.005 |
| ≥34 years | **Ref (1.0)** | 1.70 (1.04, 2.8) | 1.46 (0.91, 2.34) | 1.62 (0.85, 3.06) |  |
|  | Stable | Transition to low HDL-C | Stable | Transition to low HDL-C |  |
| Men | **Ref (1.0)** | N/A | 1.21 (0.76, 1.93) | 1.62 (0.22, 12.2) | 0.2 |
| Women | **Ref (1.0)** | 4.92 (1.07, 22.7) | 1.36 (0.69, 2.68) | 28.5 (6.35, 127) |  |
| <34 years | **Ref (1.0)** | N/A | 0.77 (0.07, 7.23) | 22.0 (1.53, 317) | 0.005 |
| ≥34 years | **Ref (1.0)** | 1.35 (0.32, 5.59) | 1.23 (0.83, 1.82) | 2.25 (0.54, 9.43) |  |

**Note:**

1. N/A, not applicable for absence of incident case of carotid artery plaque.

2. **Abbreviation**: HBP, high blood pressure; IGR, impaired glucose regulation; TC, total cholesterol; TG, total triglycerides; LDL-C, low-density lipoprotein cholesterol; HDL-C, high-density lipoprotein cholesterol.

3. **Definition**: Metabolically healthy was defined as participants without history of high blood pressure, diabetes mellitus, cardiovascular disease, dyslipidemia, and cancer but with normal blood pressure, fasting blood glucose, glycated hemoglobin A1c, TC, TG, LDL-C, and HDL-C. Dyslipidemia was defined if any of the four lipid parameters (TC, TG, LDL-C, and HDL-C) was abnormal.

4. **Criteria**: HBP (systolic blood pressure≥130 mmHg or diastolic blood pressure≥80 mmHg); IGR (FBG ≥5.6 mmol/L or glycated hemoglobin A1c≥5.7%); high TC, ≥5.72mmol/L; high TG, ≥1.7 mmol/L; high LDL-C, ≥3.4mmol/L; low HDL-C, <0.9 mmol/L in men and <1.0 mmol/L in women.

5. Model was adjusted for sex, baseline age (y), systolic blood pressure (mmHg), diastolic blood pressure (mmHg), fasting blood glucose (mmol/L), glycated hemoglobin A1c (%), TC (mmol/L), TG (mmol/L), LDL-C (mmol/L), HDL-C (mmol/L), estimated glomerular filtration rate (ml/min/1.73m^2^), and high sensitivity C-reactive protein (mg/L).

**Supplemental Table 4**. Risk of incident carotid artery plaque by baseline body weight status and transition to metabolic abnormalities: sensitivity I

| Model | Metabolically healthy normal weight  (BMI<24.0 kg/m^2^) | | Metabolically healthy overweight  (BMI≥24.0 kg/m^2^) | |
| --- | --- | --- | --- | --- |
|  | Stable | Transition to HBP | Stable | Transition to HBP |
| Participants, No (%) | 5,075 (52.1) | 2,386 (24.5) | 1,128 (11.6) | 1,158 (11.9) |
| CAP Case, No (%) | 34 (25.8) | 50 (37.9) | 14 (10.6) | 34 (25.8) |
| Multiple-adjusted model | **Ref (1.0)** | 1.73 (1.09, 2.77) | 1.22 (0.64, 2.32) | 2.37 (1.39, 4.02) |
|  | Stable | Transition to IGR | Stable | Transition to IGR |
| Participants, No (%) | 6,071 (62.3) | 1,390 (14.3) | 1,702 (17.5) | 584 (6.0) |
| CAP Case, No (%) | 55 (41.7) | 29 (22.0) | 24 (18.2) | 24 (18.2) |
| Multiple-adjusted model | **Ref (1.0)** | 1.19 (0.74, 1.91) | 1.13 (0.69, 1.86) | 1.99 (1.17, 3.37) |
|  | Stable | Transition to high TC | Stable | Transition to high TC |
| Participants, No (%) | 6,635 (68.1) | 826 (8.5) | 2,025 (20.8) | 201 (2.7) |
| CAP Case, No (%) | 60 (45.5) | 24 (18.2) | 36 (27.3) | 12 (9.1) |
| Multiple-adjusted model | **Ref (1.0)** | 2.07 (1.23, 3.48) | 1.36 (0.88, 2.09) | 2.36 (1.22, 4.57) |
|  | Stable | Transition to high TG | Stable | Transition to high TG |
| Participants, No (%) | 6,561 (67.3) | 900 (9.2) | 1,699 (17.4) | 587 (6.0) |
| CAP Case, No (%) | 68 (51.5) | 16 (12.1) | 27 (20.5) | 21 (15.9) |
| Multiple-adjusted model | **Ref (1.0)** | 1.24 (0.69, 2.23) | 1.10 (0.69, 1.75) | 2.3 (1.32, 4.02) |
|  | Stable | Transition to high LDL-C | Stable | Transition to high LDL-C |
| Participants, No (%) | 6,210 (63.7) | 1,251 (12.8) | 1,745 (17.9) | 541 (5.6) |
| CAP Case, No (%) | 50 (37.9) | 34 (25.8) | 31 (23.5) | 17 (12.9) |
| Multiple-adjusted model | **Ref (1.0)** | 1.97 (1.21, 3.22) | 1.55 (0.97, 2.49) | 2.07 (1.12, 3.8) |
|  | Stable | Transition to low HDL-C | Stable | Transition to low HDL-C |
| Participants, No (%) | 7,352 (75.4) | 109 (1.1) | 2,216 (22.7) | 70 (0.7) |
| CAP Case, No (%) | 82 (62.1) | 2 (1.5) | 45 (34.1) | 3 (2.3) |
| Multiple-adjusted model | **Ref (1.0)** | 1.63 (0.39, 6.76) | 1.29 (0.88, 1.9) | 4.65 (1.41, 15.35) |

**Note**:

1. Excluding participants whose baseline high sensitivity C-reactive protein was 10 mg/L or more (n=91)

2. **Abbreviation**: CAP, carotid artery plaque; HBP, high blood pressure; IGR, impaired glucose regulation; TC, total cholesterol; TG, total triglycerides; LDL-C, low-density lipoprotein cholesterol; HDL-C, high-density lipoprotein cholesterol.

3. **Definition**: Metabolically healthy was defined as participants without history of high blood pressure, diabetes mellitus, cardiovascular disease, dyslipidemia, and cancer but with normal blood pressure, fasting blood glucose, glycated hemoglobin A1c, TC, TG, LDL-C, and HDL-C. Dyslipidemia was defined if any of the four lipid parameters (TC, TG, LDL-C, and HDL-C) was abnormal.

4. **Criteria**: HBP (systolic blood pressure≥130 mmHg or diastolic blood pressure≥80 mmHg); IGR (FBG ≥5.6 mmol/L or glycated hemoglobin A1c≥5.7%); high TC, ≥5.72mmol/L; high TG, ≥1.7 mmol/L; high LDL-C, ≥3.4mmol/L; low HDL-C, <0.9 mmol/L in men and <1.0 mmol/L in women.

5. Model was adjusted for sex, baseline age (y), systolic blood pressure (mmHg), diastolic blood pressure (mmHg), fasting blood glucose (mmol/L), glycated hemoglobin A1c (%), TC (mmol/L), TG (mmol/L), LDL-C (mmol/L), HDL-C (mmol/L), estimated glomerular filtration rate (ml/min/1.73m^2^), and high sensitivity C-reactive protein (mg/L).

**Supplemental Table 5**. Risk of incident carotid artery plaque by baseline body weight status and transition to metabolic abnormalities: sensitivity II

| Model | Metabolically healthy normal weight  (BMI<24.0 kg/m^2^) | | Metabolically healthy overweight  (BMI≥24.0 kg/m^2^) | |
| --- | --- | --- | --- | --- |
|  | Stable | Transition to HBP | Stable | Transition to HBP |
| Participants, No (%) | 5,112 (67.0) | 834 (10.9) | 1,147 (15.0) | 542 (7.1) |
| CAP Case, No (%) | 35 (38.9) | 28 (31.1) | 14 (15.6) | 13 (14.4) |
| Multiple-adjusted model | **Ref (1.0)** | 2.63 (1.5, 4.61) | 1.29 (0.67, 2.45) | 1.94 (0.95, 3.94) |
|  | Stable | Transition to IGR | Stable | Transition to IGR |
| Participants, No (%) | 6,118 (72.6) | 375 (4.5) | 1,730 (20.5) | 201 (2.4) |
| CAP Case, No (%) | 56 (54.4) | 13 (12.6) | 24 (23.3) | 10 (9.7) |
| Multiple-adjusted model | **Ref (1.0)** | 1.03 (0.8, 2.92) | 1.12 (0.68, 1.85) | 1.9 (0.92, 3.95) |
|  | Stable | Transition to high TC | Stable | Transition to high TC |
| Participants, No (%) | 6,676 (73.5) | 272 (3.0) | 2,058 (22.7) | 79 (0.9) |
| CAP Case, No (%) | 61 (54.5) | 10 (8.9) | 36 (32.1) | 5 (4.5) |
| Multiple-adjusted model | **Ref (1.0)** | 2.22 (1.08, 4.58) | 1.37 (0.89, 2.11) | 2.66 (1.02, 6.9) |
|  | Stable | Transition to high TG | Stable | Transition to high TG |
| Participants, No (%) | 6,604 (74.5) | 298 (3.4) | 1,729 (19.5) | 239 (2.7) |
| CAP Case, No (%) | 69 (61.6) | 7 (6.3) | 27 (24.1) | 9 (8.0) |
| Multiple-adjusted model | **Ref (1.0)** | 1.68 (0.73, 3.9) | 1.1 (0.69, 1.76) | 2.39 (1.11, 5.16) |
|  | Stable | Transition to high LDL-C | Stable | Transition to high LDL-C |
| Participants, No (%) | 6,248 (72.2) | 431 (5.0) | 1,774 (20.5) | 202 (2.3) |
| CAP Case, No (%) | 51 (49.5) | 13 (12.6) | 31 (30.1) | 8 (7.8) |
| Multiple-adjusted model | **Ref (1.0)** | 1.82 (0.93, 3.57) | 1.44 (0.9, 2.32) | 2.26 (1.01, 5.03) |
|  | Stable | Transition to low HDL-C | Stable | Transition to low HDL-C |
| Participants, No (%) | 7,403 (76.1) | 50 (0.5) | 2,252 (23.1) | 30 (0.3) |
| CAP Case, No (%) | 83 (63.9) | 1 (0.8) | 45 (34.6) | 1 (0.8) |
| Multiple-adjusted model | **Ref (1.0)** | 2.01 (0.27, 14.8) | 1.24 (0.85, 1.83) | 2.60 (0.35, 19.4) |

**Note**:

1. **Abbreviation**: CAP, carotid artery plaque; HBP, high blood pressure; IGR, impaired glucose regulation; TC, total cholesterol; TG, total triglycerides; LDL-C, low-density lipoprotein cholesterol; HDL-C, high-density lipoprotein cholesterol.

2. Excluding participants who was confirmed with the following metabolic abnormalities once respectively: HBP (n=2,201), IGR (n=1,412), high TC (n=751), high TG (n=966), high LDL-C (n=1,181), low HDL-C (n=101), and dyslipidemia (n=1,793).

3. **Definition**: Metabolically healthy was defined as participants without history of high blood pressure, diabetes mellitus, cardiovascular disease, dyslipidemia, and cancer but with normal blood pressure, fasting blood glucose, glycated hemoglobin A1c, TC, TG, LDL-C, and HDL-C. Dyslipidemia was defined if any of the four lipid parameters (TC, TG, LDL-C, and HDL-C) was abnormal.

4. **Criteria**: HBP (systolic blood pressure≥130 mmHg or diastolic blood pressure≥80 mmHg); IGR (FBG ≥5.6 mmol/L or glycated hemoglobin A1c≥5.7%); high TC, ≥5.72mmol/L; high TG, ≥1.7 mmol/L; high LDL-C, ≥3.4mmol/L; low HDL-C, <0.9 mmol/L in men and <1.0 mmol/L in women.

5. Model was adjusted for sex, baseline age (y), systolic blood pressure (mmHg), diastolic blood pressure (mmHg), fasting blood glucose (mmol/L), glycated hemoglobin A1c (%), TC (mmol/L), TG (mmol/L), LDL-C (mmol/L), HDL-C (mmol/L), estimated glomerular filtration rate (ml/min/1.73m^2^), and high sensitivity C-reactive protein (mg/L).

**Supplemental Table 6**. The association between the transition to metabolically unhealthy status and the risk of incident CAP in 9,836 Chinese adults with metabolically healthy status by baseline body weight status

| Model | The number of metabolic abnormalities | | | | P trend |
| --- | --- | --- | --- | --- | --- |
|  | Stable | One type | Two types | ≥Three types | <0.001 |
| Number of Participants | 3,848 | 3,684 | 1,830 | 474 | N/A |
| CAP Case | 23 | 40 | 38 | 32 | N/A |
| Multiple-adjusted model | **Ref (1.0)** | 1.22 (0.73, 2.05) | 1.66 (0.98, 2.81) | 3.61 (2.08, 6.27) | N/A |

**Note:**

1. **Abbreviation**: **CAP**, carotid artery plaque; **HBP**, high blood pressure; **FBG**, fasting blood glucose; **HbA1c**, glycated hemoglobin A1c; **IGR**, impaired glucose regulation; **TC**, total cholesterol; **TG**, total triglycerides; **LDL-C**, low-density lipoprotein cholesterol; **HDL-C**, high-density lipoprotein cholesterol.

**2. Metabolically healthy status** was defined as no history of hypertension, diabetes mellitus, cardiovascular disease, dyslipidemia, and cancer, and normal blood pressure, FBG, HbA1c, and lipid profiles at baseline. **Metabolically unhealthy status** was defined if any of the following was confirmed during follow up: HBP (systolic blood pressure≥130 mmHg or diastolic blood pressure≥80 mmHg), or IGR (FBG ≥5.6 mmol/L or HbA1c≥5.7%), high TC, ≥5.72mmol/L; high TG, ≥1.7 mmol/L; high LDL-C, ≥3.4mmol/L; low HDL-C, <0.9 mmol/L in men and <1.0 mmol/L in women.

**3.** Multiple-adjusted model adjusted for sex, baseline age (y), systolic blood pressure (mmHg), diastolic blood pressure (mmHg), FBG (mmol/L), HbA1c (%), TC (mmol/L), TG (mmol/L), LDL-C (mmol/L), HDL-C (mmol/L), estimated glomerular filtration rate (ml/min/1.73m^2^), and high sensitivity C-reactive protein (mg/L).
